# Young men’s experiences of living with existential concerns: “living close to a bottomless darkness”

**DOI:** 10.1080/17482631.2020.1810947

**Published:** 2020-08-28

**Authors:** Maria Lundvall, Ulrica Hörberg, Lina Palmér, Gunilla Carlsson, Elisabeth Lindberg

**Affiliations:** a Faculty of Caring Science, Work Life and Social Welfare, University of Borås, Borås, Sweden; b Department of Health and Caring Sciences, Linnaeus University, Växjö, Sweden

**Keywords:** Existential concerns, reflective lifeworld research, phenomenology, qualitative research, young men, young adults

## Abstract

**Introduction:**

Young men may struggle in life with challenges of various concerns about their identity and who they want to be in life. Many health issues arise from social norms and wider societal determinations and for today’s young men, following such norms poses a risk of losing oneself. An essential part of health are connected to the existential dimensions in life and concerns who you are, and how well you know and understand yourself. However; little is known about what it means for young men to live a life with existential concerns.

**Purpose and method:**

The purpose of this phenomenological study, based on reflective lifeworld research (RLR), is to describe young men’s experiences of living with existential concerns for which they have sought support. Eight lifeworld interviews were conducted.

**Results:**

The results essentially show that young men living with existential concerns describe their situations as living close to a bottomless darkness. This is further described according to four constituents: enduring everyday life, striving for a solution, hearing an inner self-critical voice, and wearing a hard shell.

**Conclusion:**

We conclude that strengthening young men’s health processes requires healthcare professionals to create an atmosphere where young men feel safe talking about existential concerns without feeling exposed and vulnerable.

## Introduction

This study focuses on young men^1^ in Sweden living with existential concerns and is part of a larger research project studying young adults’ existential concerns.

Existence philosopher Sartre (1946/2007) claims the fundamental ontological basis for human are that some of life conditions are determined and given at birth. For example humans must relate to what has already been given to her, such as gender, heritage and culture. Humans are influenced by their past, present and future, which are intertwined and affect life. Humans’ lives also involve the freedom to choose what to do at any given moment. According to Heidegger (1927/2013), freedom involves certain limitations, such as human vulnerability, the reality of infinity or death, the fragility of our bodies, and the fact that humans live for a certain time in specific places with their own cultures and languages. Stepping into the adult world brings with it an increased vulnerability. New situations arise, which the young adult may struggle to understand. The young adults may also not understand their roles in the new situations. For some, choices and increased freedom are liberating, but for others, they cause feelings of doubt and can contribute to reflections on the meaning of life and doubts about one’s existence. In this study, existential concerns stem from the fundamental questions about life, who one is and who one wants to be as a human being. This concern might increases, during the period in life when young adults grow from being children to adults. Lundvall et al. (2019) describe when the existential concerns grow unmanageable and impair the everyday lives of young women. Existential concerns may manifest themselves in the form of mental health issues.

Studies show that the mental health issues of young adults have increased over the years, not only in Sweden but around the world (Gore et al., 2011; Socialstyrelsen, 2017). At the same time, nervousness and anxiety have increased in Sweden in young adults compared to other age groups (Lager et al., 2012). Reports and studies show differences in mental illness between young women and young men (Folkhälsomyndigheten, 2016; Hiltunen, 2017; Lager et al., 2012; Socialstyrelsen, 2017).

Research shows that young men have, to a greater extent, risky and unhealthy habits and activities that can impact their health (Mahalik et al., 2007; Statens offentliga utredningar, 2014). Research also shows that men seek care later than women do (Martínez-Hernáez et al., 2014). Another aspect of importance is how young men seeking care for existential issues are received by healthcare professionals. For example, a patient who is perceived as masculine (in psychiatry care) is more likely to be denied care, and more likely to be perceived as responsible for causing their illness themselves through, for example, aggressive behaviour and alcohol abuse (Oute et al., 2018).

Growing from a young child to an adult is a challenging time in every person’s life. During the transition to adulthood, health risks become increasingly skewed along gender lines. Young men and young women grow up under somewhat different conditions. Many health issues arise from social norms and wider societal determinations (World Health Organization, 2019). From early childhood, boys are taught to push down emotions other than anger, which can interrupt boys’ emotional development (Chaplin & Aldao, 2013). Connell (1987) describes gender as a social institution that structures relationships between men and women. Society contributes to constructing gender in a stereotypical way, for example, by giving only boy toys to boys and vice versa (Smirthwaite, 2014).

Norms in some societies describe men as robust, strong, and invulnerable; however, not displaying vulnerability can have consequences for men’s health (Yousaf et al., 2015). In many countries with a concept of hegemonic masculinity, the ideal behaviours of manhood are those which lead to control and power (Connell, 1987). Manhood is constructed, to a greater extent than womanhood, around competition, risk-taking, domination, and violence (Smirthwaite, 2014). Research has described young men as reluctant to seek help when experiencing ill-health (Jeffries & Grogan, 2012). Barriers towards help-seeking among young men include difficulty in expressing emotions, suspicion towards healthcare professionals, and not knowing where to seek support. Instead, self-medication with alcohol is one way young men may try to cope with difficult feelings (Lynch et al., 2018).

An essential part of health concerns who you are, and how well you know and understand yourself. In everyday life, young men are exposed to a variety of advice on how to look, how to live their life, and how to become a man. For today’s young men, following such advice poses a risk of losing oneself, losing one’s identity, or striving for goals that others have set rather than one’s own goals, which might increase their existential concern (Yalom, 1980). In health care, patients’ statements are often interpreted according to a medical framework, which to some extent is necessary but which is not the only possible interpretation. To grasp the true complexity of young men’s health, healthcare professionals also need to understand health in relation to the experience of being a young man. Caring sciences with a lifeworld approach provide support for describing the existential world in which all human beings find themselves; this approach unites people and interweaves them with everything else in the world. To understand how health can be improved it is important to understand the lifeworld of the person to whom the care is directed—in this study, young men with existential concerns (Dahlberg, 2018).

## Aim

The present study aims to describe young men’s lived experiences of existential concerns for which they have sought support.

## Approach and method

This phenomenological study is based on reflective lifeworld research (RLR; Dahlberg et al., 2008). RLR is founded in the continental philosophy, mainly from Husserl’s lifeworld theory (Husserl, 1936/1970) and theory of intentionality (Husserl, 1929/1977), and from Merleau-Ponty’s theories of the lived body (Merleau-Ponty, 1964/1968) and “the flesh of the world” (Merleau-Ponty, 1962/2002).

Founded in the described philosophy, the methodological principles of RLR are openness and flexibility towards, and bridling one’s own understanding of, the phenomenon being studied. These methodological principles are used throughout the research process to grasp and describe the studied phenomenon—in this study, *existential concerns* as experienced by young men.

## Participants

Eight young men between 21 and 27 years old were included in the study. They were from either a big city or a rural area in the south of Sweden. The young men had different social backgrounds and different socioeconomic circumstances. They were studying, working or on sick leave, and all were native Swedes. They had sought support from healthcare professionals, teachers, family or friends, among others. The participants were recruited in two ways:

1. Through advertising by posters on the Internet or in various centres where young men might seek support, such as primary health care, youth health centres and student health centres at high schools and universities. In these cases, the young men could contact the researcher for more information about the study.
2. Through healthcare professionals from different centres where the young men had sought support. If the participant was interested, the healthcare professional gave the participant’s phone number to the researcher, who then contacted the young man.

In both ways open questions about participating in the study were asked: Do you want to tell us about your concerns? Do you have any thoughts about the future, who you are and who you want to be as a human being? Have you sought support for your existential concern? Do you want to participate in a study to improve knowledge about existential concerns? If the young man wanted to participate, an interview took place.

## Data collection

Data were collected through lifeworld interviews (Dahlberg et al., 2008) at a place chosen by the young men, which lasted between 49 and 85 minutes (M = 56). The interviews were audiotaped and transcribed verbatim. An open and reflective approach towards the phenomenon was used during each interview. The interview started with the question “Can you describe a situation where you had experienced existential concern in life?” Thereafter, the interview continued by exploring the phenomenon through follow-up questions such as: “Can you give examples of … ?”; “What did that mean for you?”; “How did you feel when … ?”

## Data analysis

The analysis was performed in accordance with RLR principles (Dahlberg et al., 2008) and was characterized by a movement between the whole (interviews) and the parts (meanings in the data), towards a new whole (essential meaning), to describe the phenomenon’s structure of essential meanings. During the entire process of analysis, the researchers sought to keep an open mind regarding the phenomenon as well as a bridled understanding of the phenomenon. This is done to reach the essential meaning structure of the phenomenon, and its variations of meaning.

The analysis began with reading the interview transcripts several times to create a comprehensive understanding of the data material. Through openness and curiosity, the data was read to discover something new. The analysis followed by searching for meanings related to the phenomenon in focus. The meanings were related to each other and grouped into clusters based on identified similarities and differences. The creation of clusters is a dynamic process in which different possibilities are tested to search for the phenomenon’s essential structure of meanings. The essential structure is the most abstract level and describes how the meanings relate to each other. Once the essence of the phenomenon is clarified, it becomes possible to describe its constituents. The constituents describe variations and nuances of the phenomenon and together with the essence they form a whole. The constituents are described individually, and direct statements from the interviews are used to further clarify the meanings.

## Ethical considerations

This study has been guided by the research principles described in the Helsinki Declaration (World Medical Association, 2013) and the Swedish research ethics guidelines (Utbildningsdepartementet, 2003). All subjects were informed, both verbally and in writing, of the voluntary nature of the study and their right to cease participation at any time, without explanation. It was important that the participants were fully aware of the voluntary nature of their participation and that they had the right to stop participating at any time. If, after the interview, the informant expressed a need to talk about his situation, the interviewer, who is a public health nurse, stayed to continue the discussion and/or offered to set up a meeting with another healthcare professional with the ability to handle existential conversations. Ethical approval for the study was obtained from the Ethics Review Board in Gothenburg, Sweden (Dnr: 483–16), and there is an approved supplementary application for how to reach the participants (Dnr: T322-18).

## Results

The essential meaning of the phenomenon of existential concern can be described as living life close to a bottomless darkness. This bottomless darkness means that thoughts arise which question life’s meaning, and there are no obvious ways of seeing that life can change. An inner storm of emotions moves inside the body, a chaos of not recognizing oneself, and one strives for a solution to one’s life situation. This inner chaos exposes a vulnerability which reminds one of one’s weakness. Guilt and shame arise from not feeling good enough as a human being, and a self-critical and denigrating voice awakens. There is a sense of losing oneself, and one feels homeless and alienated from others. There is a struggle to handle one’s situation by wearing a hard, confident shell to prevent one from being exposed as vulnerable in the eyes of others and to hide one’s inner chaos. Time is consumed by dwelling on the negative view of one’s life situation and trying to endure life with insistent existential concerns, suffering in silence. Those suffering from existential concerns endure by just trying to live through each day while longing for a movement out of their chaos. The tension between maintaining a hard shell and one’s inner chaos slows life’s rhythm. On the one hand, the hard shell helps one get through the day, but, on the other hand, the chaos under the surface intensifies. Striving to feel at home and to belong somewhere again feels like an invincible task and leaves an empty shell of hopelessness and loneliness. Loneliness is an ambiguous feeling; sometimes aloneness is self-chosen, whereas other times it is forced. When self-chosen, it gives one the freedom to understand oneself and be at peace for a moment, but when forced, the loneliness increases the feeling of a bottomless darkness which deepens existential concerns.

The essence can be further described in terms of four constituents: enduring everyday life, striving for a solution, hearing an inner self-critical voice, and wearing a hard shell.

### Enduring everyday life

A life with existential concern is experienced as having to endure everyday life, which means there is a constant effort to distract oneself from the existential concerns so that the mind and body are occupied with other things. Existential concerns involve lingering over problems and feeling disconnected from life, which means feeling a sense of lostness regarding where one belongs: *“most of the time I kept myself busy, or I tried to harden in it and just tried to ignore it … in some way, and think that I deserve better.”*

Sometimes the young men try to endure everyday life by telling themselves to ignore the existential concerns and that they are worth more (relative to how they feel). Other times, they avoid or do not allow themselves to feel the existential concerns. This strategy may work for a while, but ultimately a sense of emptiness is experienced. The emptiness means that all feelings disappear; nothing matters and everything becomes meaningless:

everything you did still led to the same point again, you were just tired and easily irritated much of the day; you couldn’t cope with school and responsibility, you littered for example, because you couldn’t care to take the cola jar or the bottle to the garbage basket.

To endure life with existential concerns also means socializing with others to prevent the existential concerns from emerging. Being socially interactive with others and sometimes supporting others means that one forgets one’s own problems for the moment. There is a longing to be with others to distract one from the existential concerns, but at the same time being socially interactive also means that there is a risk of finding out about others’ ill-being. Others’ ill-being affects the young man negatively, creating a vicious spiral of hopelessness and a reminder of his own vulnerability:

you couldn’t cope listening to other people’s problems …. You lose your own problems and you do not see that the problems exist, it is probably what caused my depression to come, that you stayed like this, you just brushed it away.

When experiencing existential concerns, a young man can also endure everyday life by fleeing the difficult reality, passing time through keeping themselves busy. Keeping busy may mean playing computer games and fantasizing about something better—about an everyday life that is not marked by existential concern. When keeping busy to endure life, creativity and performance can also emerge. Under these circumstances, the young man performs wholeheartedly. To keep busy mostly produces a short-term sense of happiness; the existential concerns bubble beneath the surface:

if I can distract myself … then it’s nice … it comes back when I have nothing to do … because when I am alone I probably have some level of anxiety all the time … I ponder … over things.

Sometimes the young men also choose to retreat from others to endure life. To be alone is an ambiguous feeling, although it can serve as time to spend with oneself to think about life and try to figure out how to live life without feeling judged by others or risking showing vulnerability. On the other hand, when the aloneness is not chosen but forced upon the young man, this may mean that the existential concerns roam freely and the young man cannot protect himself from the strong emotions that arise in solitude:

I’ve been working on getting used to loneliness and it has worked sometimes, but sometimes it just becomes difficult, it’s like when I’m lonely then I find out that I’m lonely, and that in turn started a lot of worries.

### Hearing an inner self-critical voice

Living life as a young man experiencing existential concern means hearing an inner self-critical voice that looks for deficiencies in everything the young man does. Life seemingly moves forward, yet there is a feeling of not measuring up to what could be, and life’s rhythm feels slowed down. When one is experiencing existential concerns, guilt and shame awaken as the self-critical voice challenges thoughts of being capable in the eyes of oneself and others. Doubts about one’s value as a human being arise, and a vicious circle of self-critical thought patterns emerges: *“I felt a lot of guilt and … it’s like … I didn’t think it was easy to like myself in that situation.”*

Living with existential concerns means that there is a sense of requirement, from oneself and from society, to be someone, to do something with one’s life and to be a successful person in life. The young man may dwell excessively on failure, analysing what went wrong or whether he could have acted differently in a given situation: *“why didn’t I think about that, how could I miss that?”* Overthinking the situation and overanalysing everything becomes an obstacle that feels difficult to overcome.

When failure is experienced, questions and doubts arise about life: *“who am I if I don’t succeed?”* The young man feels that it is a weakness that he did not succeed with what he has taken on. When failures occur, negative thoughts emerge and he blames himself for the failure. To fail is to be weak, which means a vulnerability that others can take advantage of, and the young man feels a sense of shame at not being the sort of man society demands and not living up to requirements. One young man described what this feeling was like at its worst: *“a lot of pressure from family and surroundings … all the time there was a feeling that was pushing you to explore and find out for yourself who you were and what you were doing, and what to do in your life, and what you believe in.”*

Experiencing vulnerability that others can take advantage of increases the self-critical voice of not being good enough, making the young man doubt himself even more. So, to prevent anyone from seeing one’s vulnerability, a passive and more contemplative attitude is adopted, to avoid the risk of being seen as weak: *“then it was like … then I became more passive and reasoned … floated away and thought about my own things as well as I … and began … began to observe others.”*

When experiencing existential concerns, the young man watches other people’s interactions and analyses these interactions closely. Shortcomings and errors become visible in the young men’s own eyes when they socialize with others. To protect himself, the young man feels that it is easier to just be a worse version of himself, in a new relationship, than to try to win someone else’s appreciation and face the risk of not being liked by the other person, or to risk failing in the eyes of others: *“I will never be anything for this person … then I think that I have no responsibility … I can’t cope with the effort it is to say hello and how are you and so on … it might be that I am rude instead.”*

### Striving for a solution

Living life with existential concerns is experienced as striving for a solution regarding how to navigate the life situation. There is a struggle to fix what feels broken. An everyday life with existential concerns is perceived as “*a cassette tape that is played on repeat every day.”* The same thoughts about life, who you are, and who you want to be are repeated over and over again. One is constantly looking for a solution to the existential concerns, but the solution feels out of reach and the situation is seen as hopeless:

every day looked exactly alike, there were no major ups and downs but it was like a monotonous existence where there were exactly the same routines, the same problems that never really managed to change, there was no change in existence … it never got better … .

The existential concerns are always present, and a sense that there is no solution close to hand means they feel lost, unsure about who they are and who they want to be in life, and experience difficulty with managing their everyday life. Living life with existential concerns leads to a feeling of despair which is characterized by hopelessness that life will get better. The young men blame themselves for not being able to escape the darkness of their everyday life, which adds a feeling of responsibility to try to get out of the situation by themselves: *“you just felt like this; I will not manage to do this, I cannot manage to do this, and I do not know where to go; you just want to escape almost, just escape from everything and forget it.”*

Striving for a solution also means that the young man wants answers regarding how to handle life situations, with all the difficult emotions creating a sense of chaos inside him. The chaos makes the young man feel he does not recognize himself, and he feels alienated from how he used to be. The young man feels lost and broken, and endeavours to find meaning in his life and to improve his life. Striving for a solution also means that the young man seeks relations with like-minded people to feel belonging and to understand himself again. Seeking for solutions can be a lonesome process as the young man finds it difficult to talk about his existential concern with others, especially as he wants to be seen by others as a happy and positive person and does not wish to burden others with his problems: *“you need to strive to find your pack, if you want to build a solid ground to stand on. And find your own people, and I feel that’s a challenge.”*

Living with existential concerns also means that suicide is a thought that may occasionally arise as a solution to a hopeless situation, when no obvious alternatives are recognized:

I have almost been thrown, a little, against suicidal thoughts, I have felt that … why should I have to deal with this, what is the meaning of me going on and why should I suffer like this, what does it really give me?

### Wearing a hard shell

Living with existential concerns is also experienced as wearing a hard shell, which means to put on a hard, confident exterior beneath there is a sense of chaos. Existential concerns are perceived as a sensitive topic, and it feels easier not to tell others about one’s suffering. Wearing a hard shell protects the young men from show vulnerability and risking that others will exploit it, condemn them for it, or simply perceive them as vulnerable. There is an effort to find a way to keep life going between the difficult and the easy, the still and the lively, the light and the serious; the young man wants to protect himself and to not be a burden on others, by not letting others know about his existential concerns: *“I thought that no one else wants to hear about others’ problem. I never wanted to be a burden to others.”*

The hard shell can both protect and inhibit the young man. It protects the young man from being exposed as vulnerable in the eyes of others while trying to understand his life situation. At the same time, however, it can inhibit a young man’s understanding of himself to act as if nothing is wrong: *“I think it’s a bit like putting on a mask, but it’s still me. Just different sides of me. Before this in life I have only felt that I have a mask and that is the person I have been all the time, and then I never got a chance to develop the different sides of me.”*

Living with existential concerns means, for a young man, to be good at hiding these concerns to save others from perceiving these concerns and feeling bad about them. The hard shell then appears as a façade, allowing one to keep up the appearance of what one used to be, the person others recognize. The façade hides the feelings bubbling beneath the surface: *“If I walk around feeling sad and down, you become a torment to others who don’t want to be with you … you play yourself as you used to be, but you aren’t really that man inside.”*

Living with existential concerns means to display the person the young man was before and the person others recognize—a happy, positive, and self-confident person that others like. The young men find it easier to live with this happy and positive version of themselves than with the negative person they feel like and do not recognize. Keeping up the façade is easy as it means showing the version of themselves that is familiar, and it feels safe to show it. In contrast, it does not feel safe to show the new vulnerable person that they have become, and they are afraid that others will judge this new insecure version and see them as weak:

some days when you could put on this shell again, and I went to school and was my normal me and then you came home … . For me it’s that I walk around and am happy all the time but I am not, it takes so much energy and you have to fight to really keep it up.

There is a sense of relief that no one can see through the hard shell, as this means no one can see how the young man really feels. At the same time, however, a feeling of emptiness arises when one succeeds in hiding the existential concerns from close family and friends; this means that family and friends, who think they know the young man, in actuality do not see through the hard shell and do not understand the chaos that is hiding underneath:

In a way, it feels very good that not everyone really knows how I feel, but sometimes I can feel very empty from it … because I know they do not know me as well as they think they do.

## Discussion

The purpose of this study is to describe young men’s experience of living life with existential concerns. The results show that young men with existential concerns live a life in which they suffer in silence. The young men strive for solutions to their life situation, and a feeling of being stuck in limbo emerges. Daily life is endured by trying to get through one day at a time, and life is overshadowed by a bottomless darkness.

The fact that the young men suffer in silence when experiencing existential concerns can be related to Galvin and Todres (2013) description of suffering that emphasizes a sense of temporary stagnation in which the individual feels “stuck”; they feel blocked and the future seems uncertain. Their lack of temporary mobility means that not much about the future seems attractive. A sense of meaninglessness or lack of purpose manifests, and the present is reduced to oppressive repetition. In the worst case, a person may feel that life is slowing down and a feeling of being stuck in time will arise. Not much tempts one to move forward, towards the future. This reasoning is in line with the results of the present study, whereas the young man, when experiencing existential concerns creates a sense that life’s rhythm is slowed down and a sense of hopelessness about one’s life situation.

Galvin and Todres (2013) further describes a temporal dimension of dwelling-mobility suffering; in other words, the temporal qualities of a blocked future and an elusive present can be seen as intertwined. The most intensive form of temporal suffering occurs when the “present is intolerable and the future is repellent” (Galvin & Todres, 2013, p. 102). This means that neither the present nor the future seems to present any opportunities for respite. From this, we can understand that young men experiencing existential concerns live close to a bottomless darkness. According to Galvin and Todres (2013), in such a situation, healthcare professionals not only need to fully acknowledge a young man’s expression of this bottomless darkness and of their lack of respite but also must assist these young men in their search for a way out of their bottomless darkness. At the same time, life’s rhythm slows, and life seems meaningless and hopeless. The results show that the young men’s strategy to endure everyday life consists of being constantly active or engaged. Being active is necessary to distract oneself from existential concerns and to avoid feeling unwell. According to Martínez-Hernáez et al. (2014), young men with symptoms of depression try to forget about their trouble by engaging in activities with others and by trying to normalize their symptoms. To forget their problems for a while is a prerequisite to feeling like it is possible to talk about their problems.

Awakening of existential concerns challenges our basic assumptions about life (Yalom, 1980), and to avoid being trapped in meaninglessness, it is up to each individual to find their own subjective meaning in a world in which there is no obvious purpose to life (Udo, 2014). Yalom (1980) indicates it is difficult to avoid this sense of meaninglessness. The present results show that young men put on a hard, confident shell to protect themselves from others’ judgemental attitude. The hard shell lets a young man play a role, as what others expect him to be, to play the social game and be “normal.” The young men hide their authentic selves and feel unwell under this hard shell.

The results show that young men experiencing existential concerns suffer in silence and try to handle their life situation by themselves to avoid appearing vulnerable. This is in line with the work of Danielsson et al. (2011) who found that young men with depression seldom tell others how they feel, and that the young men adopt a “Superman” heroic appearance to deal with their life situation while mulling over their problems alone. The young men feel it is difficult and shameful to let anyone else know that they are sad and feeling down. They struggle to be normal. Striving for normalcy is not done to challenge societal norms, but in fact confirms prevailing norms (Danielsson et al., 2011).

The study’s results also show that young men feel alone with their existential concerns, and this aloneness sometimes is self-chosen but sometimes feels forced. This loneliness is also explored by Yalom (1980), who describes existential isolation as different from social isolation. Existential isolation is characterized by the vulnerability of our existence, meaning that we begin life alone and we leave this life alone. With this in mind we can deduce from our results that the young men feel existentially isolated.

Furthermore, Martínez-Hernáez et al. (2014) found that young men see their problems as too personal to share with someone else. To seek support is seen as a weakness that challenges the future view of oneself as an autonomous and independent individual. Bolmsjö et al. (2019) state that existential loneliness can be understood “as the immediate awareness of being fundamentally separate from other people and from the universe” (Bolmsjö et al., 2019, p. 12), when an individual does not have anybody to communicate with in a deeper human way (on an authentic level). This can lead to negative emotions, for example hopelessness and meaninglessness (Bolmsjö et al., 2019).

According to Lundvall et al. (2018), healthcare professionals’ own existential concerns are awakened by their conversations with young adults expressing existential concerns. Bullington et al. (2019) finds that healthcare professionals’ own personal fears and definitions may create difficulties in conversation, and therefore conversations of this type are not prioritized. Furthermore, they suggest using phenomenologically based communication to elucidate a person’s own experience of ill-being. Lundvall et al. (2019) describe the importance, when meeting young women with existential concerns in their lifeworld, of creating a caring relationship so that the young women feel safe and are willing to talk about their innermost thoughts. From the results of the present study we see the importance for healthcare professionals to be aware of dominant masculine ideals that young men live with, and that healthcare professionals must also be aware of their own potential prejudices when attempting to understand the needs of young men (Oute et al., 2018) seeking care for their existential concerns.

The purpose of health care is to strengthen patients’ health processes. Health involves the whole person and a sense of balance in relation to life and people around. From a caring sciences perspective, it is important for healthcare professionals to understand the suffering of their patients—in this case young men experiencing existential concerns—to be able to support health processes towards well-being (Dahlberg, 2011). When healthcare professionals must deal with young men with existential concerns, an important starting point is to try to understand the lifeworld of these young men (Dahlberg, 2018). According to Toombs (2002), understanding the “insider” perspective is a pivotal concern, particularly in any field where the human activity of “caring” is important. Todres et al. (2014) argue that when healthcare professionals try to understand the “insiderness” of their patients, the important thing is not to have all the “right” answers and fully understand the content of the “insiderness,” but rather to understand the vulnerability that the patients feel in the moment. It is important for healthcare professionals to understand that they hold great power in being a person who creates trust, rather than being a person “who knows everything.”

With a lifeworld-led approach, there is a shift of understanding away from a personal expectation of certainty to “the reaching” towards the “ambiguous” human situation (Todres et al., 2014). A lifeworld perspective requires interpersonal meetings, which take place within the framework of a caring relationship which has the potential to point to a direction for care. Lifeworld-led care focuses not only on the absence of illness but also on well-being. The concept of well-being includes an existential dimension as well as vulnerability and freedom. There is a risk that these dimensions will be overlooked if the care is only directed towards the absence of illness (Galvin & Todres, 2011, 2013).

## Methodological reflections

This phenomenological study uses an RLR approach, following Dahlberg et al. (2008), to describe the experience of existential concerns. The whole research process has been guided by the RLR methodological approach, in terms of openness, bridling, and a reflective attitude towards the phenomenon to achieve the study objective and avoid taking for granted what is unknown during the analysis. This was accomplished by continually asking reflective critical questions to slow down the understanding process. Questions such as “Why do we understand it like this?” and “Could it mean something else?” guided the research process. One difficulty with maintaining objectivity in interviews is to keep the interview alive and focused on the phenomenon; here the interviewer stayed close to what the informant tells, yet still maintained a distance to what is told so as not to miss any nuance of the phenomenon. The interviewer had to go slowly in the interview and not hast with follow-up questions. One limitation, from a positivistic view, is that the low number of participants might be seen as a hindrance to generalizing the results.

From a phenomenological point of view, the strength of this study lies in the depth and richness of the description of the phenomenon rather than the number of participants (Dahlberg, 2019). We think that the variety of participants contributed to revealing rich nuances of the phenomenon and permitted us to describe its essence and its constituents, providing validity to the study. To talk about generalizability we must always keep in mind that results are contextual. Since the results are presented as an abstract essence and are based on a variety of data, there is reason to believe the present results are transferable to similar contexts.

## Conclusions and implications

This study provides important knowledge from a caring science perspective which can contribute to a caring approach in the nursing practice. Young men experiencing existential concerns perceive themselves as existing close to a bottomless darkness in which they alone search for a solution to their life situation. The search for a solution is always ongoing, and the young men want to feel normal and at home again. To strengthen the health process for a young man with existential concerns, we suggest that healthcare professionals (such as nurses in youth centres, student healthcare centres, primary healthcare centres and other centres where they meet young men) practise lifeworld-led care. Lifeworld-led care has the potential to reveal the existential dimension of young men’s vulnerability. Furthermore, it is of utmost importance for healthcare professionals to create an atmosphere in which a young man can find a place to dwell, as a man, on his innermost thoughts without feeling vulnerable so that he can improve his well-being.

## Note

1. In this article, we refer to young men, and young adults in general, as those from 16 to 25 years of age. References to other ages are given in parentheses.

## Additional note

The article is the authors’ original work, has not received prior publication and is not under consideration for publication elsewhere.

## Permission note

All material in the manuscript is original content.
